# A rare transitory change of the De Winter ST/T-wave complex in a patient with cardiac arrest: A case report

**DOI:** 10.1097/MD.0000000000020133

**Published:** 2020-05-08

**Authors:** Li Liu, Dan Wang

**Affiliations:** a Department of Cardiology of Hankou District of the General Hospital of Central Theatre Command of PLA; b Department of Ophthalmology of the General Hospital of Central Theatre Command of PLA, China.

**Keywords:** De Winter ST/T-wave complex, electrocardiogram, ST elevation myocardial infarction

## Abstract

**Rationale:**

The De Winter ST/T-wave complex is a rare and special electrocardiogram (ECG) manifestation in some patients with a total or subtotal occlusion in the proximal left anterior descending (LAD) coronary artery. It mainly appears as an ST-segment superior oblique depression instead of an ST elevation. However, a transitory change of the De Winter ST/T-wave complex has not been reported previously.

**Patient concerns:**

A 40-year-old man developed sudden precordial dull and unrelieved pain. One hour later, he suddenly lost consciousness when he arrived at the emergency department. After successful cardiopulmonary resuscitation (CPR), 2 ECGs were taken at 22-minute interval, which showed completely different manifestations. The first ECG showed acute inferior-wall ST elevation myocardial infarction (STEMI), while the second ECG showed a De Winter ST/T-wave complex, which indicated acute anterior-wall myocardial infarction.

**Diagnosis:**

The patient was diagnosed with acute myocardial infarction.

**Interventions:**

The patient responded to urgent treatment by percutaneous coronary intervention (PCI).

**Outcomes:**

It was confirmed that the case was consistent with the main characteristics of a De Winter ST/T-wave complex after PCI. The first ECG was a rare transitory change of the De Winter ST/T-wave complex. The patient was well recovered and discharged.

**Lessons:**

The De Winter ST/T-wave complex is an extremely dangerous and rare ECG manifestation that is not widely recognized at present. Although the mechanism is not very clear, it should be considered as indicating an equivalent risk of STEMI because it may suggest total or subtotal occlusion in the proximal LAD coronary artery. It is believed that in the near future, the mechanism of ECG including its transitory changes, will be fully revealed.

## 1 Introduction

ST elevation myocardial infarction (STEMI) is an acute occlusion of the coronary artery caused by coronary plaque injury. It gives rise to myocardial ischemia, hypoxia, and necrosis, which can result in corresponding clinical symptoms.^[1]^ The super-acute phase electrocardiogram (ECG) of STEMI often shows an ST elevation and T-wave high point in the related leads. However, in 2008, De Winter et al described an ST-segment superior oblique depression and T-wave high point on the chest lead ECG of some patients with total or subtotal occlusion in the proximal left anterior descending (LAD) coronary artery. It was named the De Winter ST/T-wave complex. Recently, we encountered a patient with cardiac arrest who responded positively to cardiopulmonary resuscitation (CPR). The patient's early ECG showed acute inferior-wall STEMI. After 22 minutes, the ECG changed into a De Winter ST/T-wave complex. However, whether the real cause of cardiac arrest was acute inferior or anterior wall myocardial infarction remains unclear. The patient had provided informed consent for publication of this case. The present study was approved by the Ethics Committee of Hankou District of the General Hospital of Central Theatre Command of PLA.

## 2 Case report

**Figure 1:**
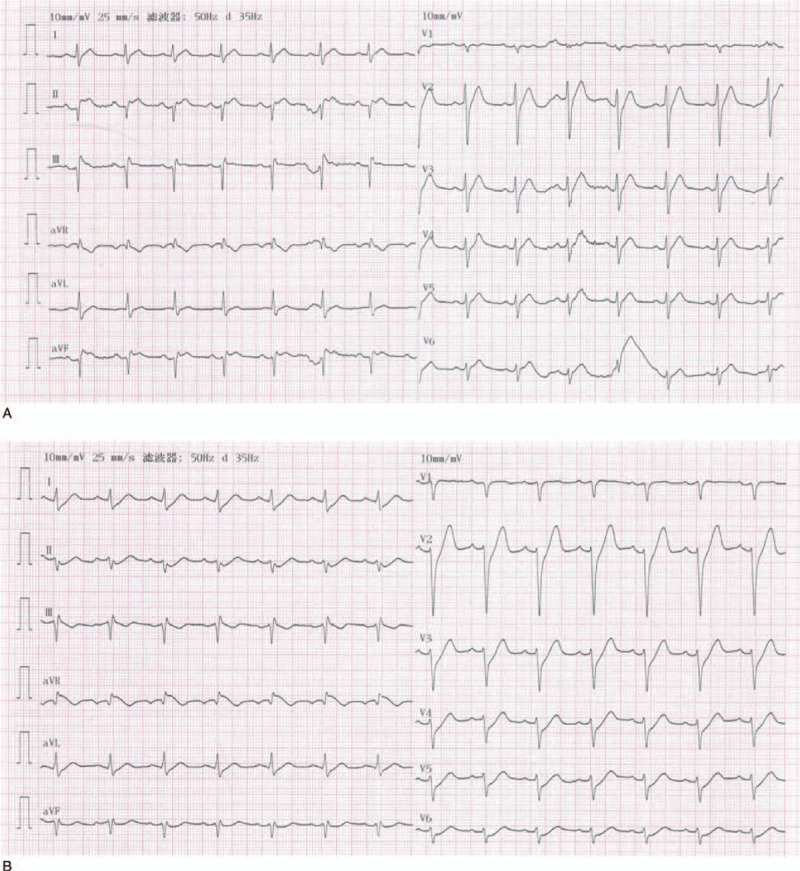
(A) ECG before percutaneous coronary intervention at 18:47 on April 28, 2019. (B) ECG before percutaneous coronary intervention at 19:09 on April 28, 2019.

**Figure 2:**
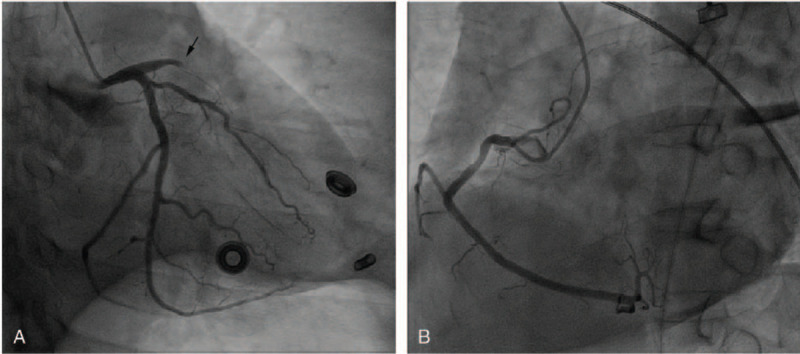
(A) Coronary angiography for left coronary artery (RAO30°+CAU30°). (B) Coronary angiography for right coronary artery (LAO45°).

**Figure 3:**
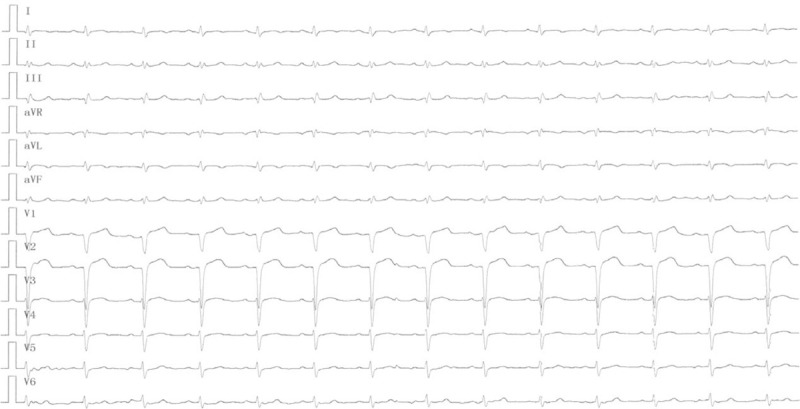
ECG after percutaneous coronary intervention.

A 40-year-old man developed sudden precordial dull and unrelieved pain accompanied by sweating for 1 hour. When he arrived at the emergency department of our hospital at 18:30 on April 28, 2019, he suddenly lost consciousness with halted breathing and cardiac arrest. The doctors urgently performed CPR on him, including chest compressions, electric defibrillation 3 times, tracheal intubation, ventilator assisted breathing, and dopamine intravenous infusion. After about 10 minutes, his respiration and palpitation recovered, but he was still unconscious with the double pupil dilation of 7 mm. His vital signs revealed a body temperature of 36.2°C, a heart rate of 89 beats per minute, a respiration rate of 32 breaths per minute, and a blood pressure of 95/52 mmHg. The patient had a history of hyperlipidemia for 1 year. The first ECG at 18:47 showed a sinus rhythm with 89 beats per minute, and ST-segment elevation 1mv in leadsII and aVF, and 0.5 mv in leadIII (Fig. 1A). This suggested that the patient had acute inferior-wall STEMI, and the infarct-related artery was located in the right coronary artery or the left circumflex coronary artery. Percutaneous coronary intervention (PCI) was proposed for emergency treatment. The second ECG at 19:09 showed an sinus rhythm with 83 beats per minute, ST-segment restoring to a near baseline level in leadsII, III, and aVF, ST-segment superior oblique depression (J-point amplitude depression 1–5mv) in leadsV2-V6, ST-segment superior oblique depression (J-point amplitude depression 1–2mv) in leadsI and aVL, ST-segment elevation 1.5 mv in leadaVR, poor R-wave rising in leadsV1-V6, and T-wave high point and symmetry in leadsV2 and V3 (Fig. 1B). The ECG changed to a De Winter ST/T-wave complex, which suggested that the patient developed acute anterior-wall myocardial infarction, and the infarct-related artery was located in the proximal LAD coronary artery. The 2 ECGs, which were 22 minutes apart, showed significant differences that revealed completely different infarct-related arteries. It was unclear which one revealed the actual infarct-related artery. We urgently treated the patient with PCI. Coronary angiography showed a normal left main coronary artery, mild stenosis in the mid-segmental left circumflex coronary artery, acute total occlusion in the proximal LAD coronary artery (Fig. 2A), and mild stenosis in the proximal right coronary artery (Fig. 2B). At this point, the diagnosis was clear. Stent implantation was successfully performed in the proximal LAD coronary artery. Postoperative ECG showed an sinus rhythm with 79 beats per minute, the QRS-wave was QS type in leadsV1 and V2, ST-segment restoring to a near baseline level in leadsV1-V6 (ST-segment elevation 0.05–0.15 mv in leadsV1 and V2), the T-wave was lower, and the limb lead voltage was low (Fig. 3). Partial preoperative blood biochemical results showed high-sensitivity troponin I (hs-TNI) level of 279.1 pg/mL (range, 0–26.2 pg/mL), cholesterol level of 9.16 mmol/L (range, 3.12–5.69 mmol/L), triglyceride level of 1.90 mmol/L (range, 0.48–1.71 mmol/L), low density lipoprotein cholesterol level of 7.14 mmol/L (range, 2.07–3.40 mmol/L), and high density lipoprotein cholesterol level of 0.96 mmol/L (range, 0.78–2.00 mmol/L). The reexamination blood biochemical results on April 29, 2019 showed hs-TNI level of 39,779.4 pg/mL. The patient regained complete consciousness on April 30, 2019. The patient recovered well after PCI and was discharged from the hospital on May 13, 2019.

## 3 Discussion

De Winter et al first described the De Winter ST/T-wave complex in 2008.^[2]^ The ECG suggested a total or subtotal occlusion in the proximal LAD coronary artery. Its characteristics were as follows:

(1) The ST-segment of leadsV1-V6 was a superior oblique depression with J-point amplitude depression≥1mv. The T-wave was a high point and symmetrical.
(2) The QRS-wave time limit was normal or slightly prolonged.
(3) A part of the ECGs had poor R-wave rising in the chest lead.
(4) Most ECGs had slight ST-segment elevation in leadaVR.

This ECG manifestation was static and could not be displayed as STEMI.^[3]^ In the present case, the characteristics of the second ECG were completely consistent with a De Winter ST/T-wave complex. It was confirmed that the case was consistent with the main characteristics of a De Winter ST/T-wave complex, and the lesion was located in the proximal LAD coronary artery by subsequent PCI. Verouden et al^[4]^ found that De Winter ST/T-wave complexes occurred in 35 of 1890 patients with acute anterior-wall myocardial infarction, with an incidence of <2.0%. Compared with patients with acute anterior-wall STEMI, most patients with a De Winter ST/T-wave complex were men with hypercholesterolemia. The patient in the present report was a middle-aged man whose cholesterol and low density lipoprotein cholesterol level were significantly higher than normal. This was also consistent with the characteristics of a patient with De Winter ST/T-wave complex.

In the present case report, the patient was critically ill and his condition deteriorated to sudden cardiac arrest 1 hour after the onset of chest pain. Successful CPR was performed. Interestingly, the transitory ST-segment elevation in leadsII, III and aVF was recorded in the first ECG, which led to the incorrect diagnosis of acute inferior-wall STEMI. Then, the second ECG rapidly changed into a De Winter ST/T-wave complex. This change has not been previously reported. Whether the ECG manifestation was related to cardiac arrest, ED, and CPR or the normal transitory change of the De Winter ST/T-wave complex was not very clear. Until now, there have been many speculations about the mechanism of De Winter ST/T-wave complexes. It is speculated that this may be due to severe subendocardial ischemia, which results in baseline elevation and relative ST-segment downward movement, or repeated myocardial ischemia, which results in a wide range of collateral circulation between coronary arteries.^[5,6]^ The subendocardial repolarization delay accompanied by the formation of a transmembrane action potential results in a T-wave high point and ST-segment superior oblique depression. The transmembrane action potential has the characteristics of a slow rise period and long duration. An additional minor change in transmembrane action potential exerted on the epicardium results in a J-point amplitude depression and T-wave high point.^[7,8,9]^ It is also believed that the ECG will show a high and wide T-wave when the blood supply to the heart decreases, intracellular potassium ion outflow and repolarization delays.^[10,11]^ We speculate that the cause of the ECG in this case showing a transitory ST-segment elevation in leads II, III, and aVF may be related to electric defibrillation and CPR. The rescue process affects the potential of local myocardial. When the potential difference between the endocardial and epicardial changes, the ECG will present an ST-segment elevation, which falsely suggests STEMI. After the potential recovers and the difference disappears, the ST-segment elevation will return to baseline. Therefore, ST-segment elevation is only a transitory phenomenon. In addition, in the early stage of successful cardiac resuscitation, the systolic and diastolic functions of the right ventricular myocardium are worse than those of the left ventricular myocardium. This may lead to insufficient perfusion of the right coronary artery because myocardial diastole is the driving force of coronary perfusion. Therefore, the first ECG showed acute inferior-wall myocardial infarction. The above mechanism is only our speculation, and the real reasons need to be further explored.

In clinical work, the De Winter ST/T-wave complex is an extremely dangerous ECG manifestation. This may suggest total or subtotal occlusion in the proximal LAD coronary artery. Clinicians should pay increased attention to patients with a high risk of sudden cardiac arrest. Although many experts believe that the De Winter ST/T-wave complex should be treated as indicating an equivalent risk of STEMI,^[12,13]^ this ECG manifestation is not mentioned in either the 2012 ACCF/AHA non-STEMI guidelines^[14]^ or 2013 ACCF/AHA STEMI guidelines.^[15]^ In addition, this ECG manifestation has not been mentioned in the fourth universal definition of myocardial infarction.^[1]^ This is because the De Winter ST/T-wave complex is extremely rare and not widely recognized at present. Its occurrence mechanism is unknown, so it cannot be clearly classified as non-STEMI or STEMI. However, it is believed that the De Winter ST/T-wave complex will attract more attention in the near future. Its definition, classification, mechanism, and treatment will be completely understood and expounded. At that time, the mechanism of ECG transitory changes described in this article will also be fully revealed.

## Author contributions

**Data curation:** Dan Wang.

**Formal analysis:** Li Liu.

**Investigation:** Li Liu.

**Methodology:** Li Liu.

**Project administration:** Li Liu.

**Resources:** Li Liu.

**Supervision:** Dan Wang.

**Validation:** Dan Wang.

**Visualization:** Dan Wang.

**Writing – original draft:** Li Liu.

**Writing – review & editing:** Li Liu.
